# Monocyte-to-high density lipoprotein ratio is associated with a decreased compound muscle action potential amplitude in patients with diabetic axonal polyneuropathy

**DOI:** 10.1097/MD.0000000000012857

**Published:** 2018-10-19

**Authors:** Gönül Vural, Şadiye Gümüsyayla

**Affiliations:** Department of Neurology, School of Medicine, Yıldırım Beyazıt University, Ankara, Turkey.

**Keywords:** axonal polyneuropathy, diabetic polyneuropathy, monocyte/high-density lipoprotein ratio, oxidative stress

## Abstract

The monocyte-to-high density lipoprotein ratio (MHR) has recently been implemented as an indicator of inflammation and oxidative stress. The present study characterized MHR in patients with diabetic polyneuropathy (DPN), in which oxidative stress and microvascular damage play a role in pathogenesis, relative to patients with non-DPN, diabetic patients without polyneuropathy, and healthy individuals. We further aimed to evaluate the association between MHR and the decreased compound muscle action potential (CMAP) amplitude of patients with diabetic axonal polyneuropathy.

We enrolled 90 patients with DPN, 75 patients with nonDPN, 92 diabetic patients without polyneuropathy, and 67 healthy individuals; The monocyte, high-density lipoprotein cholesterol (HDL-C) values were obtained for all participants and MHR was calculated for each individual. Intergroup comparison was performed. The relationship between MHR and the posterior tibial nerve CMAP amplitudes was examined.

Statistically significant negative correlation was observed between MHR and the posterior tibial nerve CMAP amplitudes of patients with DPN. The MHR values of the patients with DPN were significantly higher than those of the patients with non-DPN, diabetic patients without polyneuropathy and the control group.

This study demonstrated that diabetic patients with higher MHR values may be more likely to develop polyneuropathy.

## 1 Introduction

Diabetic neuropathy is one of the most common causes of peripheral neuropathies and is a major cause of low quality of life and morbidity in patients with diabetes.^[1]^ The most commonly observed type is the length-dependent pattern of distal symmetric polyneuropathy, which is associated with progressive distal axonopathy.^[2]^ Although the cause of diabetic polyneuropathy (DPN) is not known, metabolic and ischemic causes, autoimmune factors, oxidative stress, and inflammation account for its etiology.^[1,3–6]^

The monocyte/high-density lipoprotein ratio (MHR) indicates inflammation and oxidative stress due to the proinflammatory effect of the monocytes, as well as the anti-inflammatory and antioxidant effect of the high-density lipoprotein cholesterol (HDL-C). Several studies have used these metrics to determine whether inflammation and atherosclerosis contribute to the etiopathogenesis of cardiovascular and cerebrovascular diseases.^[7–14]^ However, such indicators have not been employed by research to elucidate the cause of DPN, despite ischemia, and inflammation frequently referenced as factors in its etiopathogenesis.

The present study sought to address this dearth in the literature by investigating the relationship between the monocyte/high-density lipoprotein ratio and the decreased compound muscle action potential (CMAP) amplitude in patients with diabetic axonal polyneuropathy.

## 2 Materials and methods

### 2.1 Study design and patients

We retrospectively analyzed the consecutive electroneuromyography (ENMG) results of patients who were referred to our electrophysiology laboratory for electrodiagnostic examination from January 2017 to June 2018 because of the following clinical symptoms and signs that suggested polyneuropathy: pain, numbness, tingling, ataxia, muscle weakness, autonomic symptoms, reflex loss, and loss of sensation. Ethical approval was not necessary because this was a retrospective study.

The medical records and neurologists’ examination notes of diabetic patients whose electrodiagnostic examinations were normal, as well as those of all diabetic and nondiabetic patients with electrophysiologically diagnosed axonal polyneuropathy, were evaluated to determine the etiological cause of polyneuropathy. The patients were classified into 2 groups for analysis according to whether they had been previously diagnosed with diabetes or not. The patients with diabetes mellitus who were referred for suspected polyneuropathy were further subdivided into 2 groups according to whether or not they had electrophysiologically axonal polyneuropathy: patients with DPN and diabetic patients without polyneuropathy. Non-diabetic patients with axonal polyneuropathy were classified into a separate group. This group consisted of the following:

(1) the patients who attended follow up due to a diagnosis of chronic renal failure had sub-clinical neuropathy screening for hemodialysis indication, or who were diagnosed with possible uremic polyneuropathy,
(2) patients who had continuously consumed too much alcohol and who were subsequently diagnosed with alcoholic polyneuropathy after having been referred to us for having demonstrated clinical findings of polyneuropathy,
(3) patients who were examined due to the neuropathic complaints that started when they received chemotherapy for cancer and were considered to have polyneuropathy associated with a chemotherapeutic agent,
(4) patients who had an uncertain etiology of polyneuropathy and who were consequently diagnosed with idiopathic polyneuropathy. Patients who were diagnosed with axonal polyneuropathy as a result of electrodiagnostic examination but who featured comorbidities that could account for their polyneuropathy and patients with newly diagnosed diabetes were excluded from the study. The healthy controls did not have diabetes or any other chronic disease, nor did they have any symptoms or signs associated with polyneuropathy.

### 2.2 Demographic, medical, and laboratory data

Participants’ medical records were reviewed and the following clinical data were collected: age, gender, duration of diabetes mellitus, insulin use, history of hypertension, statins use, previous medical history. Peripheral venous blood samples of the patients were collected from veins at the time of their admission to the outpatient clinic. Peripheral blood count parameters, the percentage of glycosylated Hemoglobin A1c (HbA1c), total cholesterol (TC), low-density lipoprotein-cholesterol (LDL-C), HDL-C, and triglyceride (TG) levels were recorded. The MHR was calculated for each individual by dividing their monocyte count with their HDL cholesterol level.

### 2.3 Electrophysiology and axonal polyneuropathy diagnosis

Motor and sensory conduction studies were performed in all patients following receipt of approval for testing. Sensory transmission studies were conducted in the unilateral median, ulnar, and sural nerves. Motor nerve transmission studies were performed in the unilateral median and ulnar nerves, and the bilateral posterior tibial and peroneal nerves by using a Keypoint electromyography (EMG) machine. Observation of decreased action potential amplitudes in 2 or more nerves without the existence of criteria for demyelination, such as a significant slowdown in the nerve conduction rates or conduction blocks, was indicative of axonal polyneuropathy.

Intergroup comparison was performed. The relationship between the posterior tibial nerve CMAP amplitudes and the aforementioned parameters was examined. The posterior tibial nerve was selected for analysis because the majority of polyneuropathies are length dependent. The extensor digiti brevis muscle was misleading for measuring the fibular nerve amplitude due to atrophy in the years due to the shoe pressure, and the sural nerve amplitude decreased with age.

### 2.4 Statistical analysis

SPSS 20 (IBM Corp. Released 2011. IBM SPSS Statistics for Windows, Version 20.0. Armonk, NY: IBM Corp.) statistics software was used for the evaluation of the data. The mean ± standard deviation, number, and percentage values were used as the variables. The homogeneity of the variances, which is one of the prerequisites of the parametric tests, was checked with the “Levene” test. The normality hypothesis was checked with the “Shapiro-Wilk” test. To identify differences between the 2 groups, the Student *t* test was used when parametric tests satisfied the pre-conditions, and the Mann–Whitney *U* test was used when parametric tests did not. One-way analysis of variance and Tukey honestly significant difference test were used for comparisons of 3 or more groups. “Kruskal Wallis” and “Bonferroni-Dunn” tests were used when the parametric test prerequisites were not fulfilled. The relationship between 2 variables was evaluated by Pearson correlation coefficient when parametric tests satisfied the pre-conditions and by Spearman correlation coefficient when parametric tests did not fulfill the pre-conditions. The relationships between categorical variables were analyzed using the Chi-square test and Fisher exact test. The Monte Carlo simulation method was used when the expected frequency values were smaller than 20% for inclusion of these frequencies in the analysis. Univariate and multivariate logistic regression analyses were used to characterize the relationship between independent and dependent variables (diabetic patients with and without polyneuropathy) and to present multivariate risk factors. The statistical significance level was set to *P* < .05 and *P* < .01.

## 3 Findings

The present study included 90 patients with DPN, 75 patients with non-DPN, 92 diabetic patients without polyneuropathy, and 67 healthy individuals. The basic characteristics and laboratory results concerning the patients are shown in Table 1. The average disease duration of patients with DPN was significantly longer than that of diabetic patients without polyneuropathy (*P* = .040). Relative to diabetic patients without polyneuropathy, DPN patients featured higher HbA1c values, as well as insulin and statin use rates **(***P* = .001, *P* = .003, *P* = .028, respectively) (Table 2). While the mean posterior tibial nerve CMAP amplitude of patients with DPN was 1.55 mv (0.1–5 mv), the mean posterior tibial nerve CMAP amplitude of patients with non-DPN was 2.04 mv (0.1–5 mv). Of the 75 patients who did not have diabetes, 35 had chronic renal failure, 9 chronically used excessive alcohol for more than 18 years, 27 patients received treatment with antineoplastic chemotherapeutic agents due to various cancers, and 5 patients were diagnosed with idiopathic polyneuropathy.

**Table 1:**
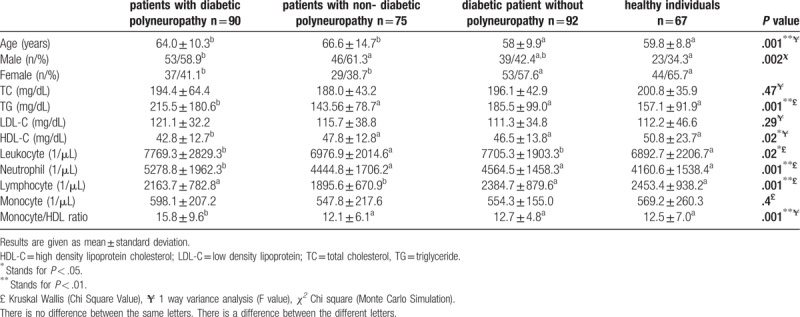
The characteristics, lipid profiles, and peripheral blood count parameters of the patients and healthy individuals.

**Table 2:**
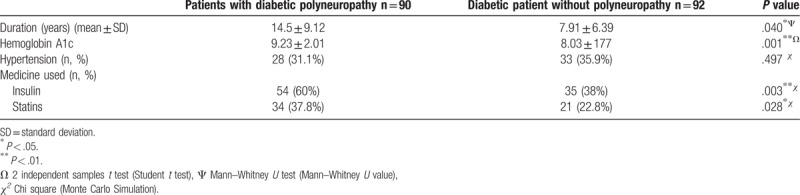
Characteristics of diabetic patients with and without polyneuropathy.

TG levels of the patients with DPN were significantly higher than those of the non-DPN group, diabetic patients without polyneuropathy, and healthy individuals (*P* = .001). There was no significant difference between the groups in terms of the LDL-C and TC levels (*P* = .29 and *P* = .47, respectively). HDL-C levels of the patients with DPN were significantly low compared to those in the non-DPN group, diabetic patients without polyneuropathy, and the control group (*P* = .02). While there was no significant difference between the groups in terms of monocyte count, neutrophil counts were higher in the patients with DPN relative to the patients in the 3 other groups (*P* = .4, *P* = .001, respectively). Lymphocyte counts of the patients with non-DPN were significantly lower than those of all other patients (*P* = .001) (Table 1). MHR was not different among the patients with non-DPN, diabetic patients without polyneuropathy, and healthy individuals. The MHR values of the patients with DPN were significantly higher than those of the patients with non-DPN, diabetic patients without polyneuropathy, and the control group (*P* = .001) (Table 1).

In the univariate analysis, age, sex, MHR, neutrophil, HbA1c, disease duration, insulin use, and statin use were demonstrated to be possible confounding factors for the polyneuropathy. After multivariate logistic regression analysis, age, MHR, neutrophil, HbA1c, and disease duration emerged as independent predictors of DPN. Univariate and multiple logistic regression analysis indicated that age (OR = 1.054, 95% CI = 1.008–1.101, *P* = .020), MHR (OR = 1.222, 95% CI = 1.017–1.470, *P* = .033), neutrophil (OR = 1.000, 95% CI = 1.000–1.001, *P* = .018), HbA1c (OR = 1.373, 95% CI = 1.087–1.733, *P* = .008) and disease duration (OR = 1.105, 95% CI = 1.040–1.175, *P* = .001) are independent predictors of DPN (Table 3).

**Table 3:**
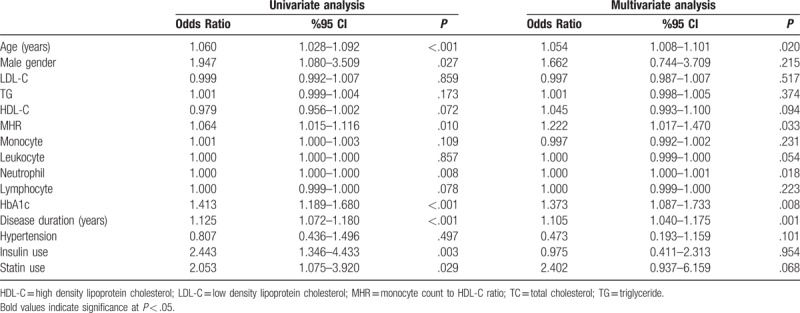
Univariate and multivariate logistic regression analyses performed to identify possible confounding factors of diabetic polyneuropathy.

No correlation was observed between the levels of TC, TG, LDL-C, the counts of leukocyte neutrophils, lymphocytes, HbA1c, and the posterior tibial nerve CMAP amplitudes in the patients with DPN. There was a strong positive correlation between the posterior tibial nerve CMAP amplitudes and HDL-C values (*P* = .000, r = .360). The decrease in the posterior tibial nerve CMAP amplitudes was negatively correlated with the increase in both monocyte counts and MHR (*P* = .016, r = -.253; *P* = .008, r = -.279, respectively) (Table 4).

**Table 4:**
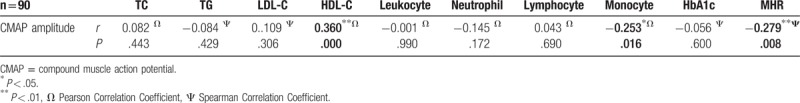
The relationship between lipid profile and peripheral blood count parameters of patients with diabetic polyneuropathy and their posterior tibial nerve compound muscle action potential amplitudes.

## 4 Discussion

The present study demonstrated that the increase in MHR levels of patients with diabetic axonal neuropathy was correlated with the decrease of the CMAP amplitude. To the best of our knowledge, this is the first study that demonstrates patients diabetic axonal neuropathy with exhibiting higher values of MHR relative to diabetic patients without polyneuropathy, with nondiabetic axonal neuropathy, and healthy controls.

Neuropathy is the most common microvascular complication of diabetes mellitus, and distal peripheral neuropathy is the most frequently observed form of neuropathy in patients with diabetes.^[15]^ Despite being one of the major causes of morbidity in patients with diabetes,^[16–18]^ prior research has not investigated it as and the mechanisms underlying DPN have yet to be elucidated. Monocyte-to-high density lipoprotein ratio (MHR) has recently been implemented as an indicator of inflammation and oxidative stress. The present study determined how MHR changed in patients with DPN, in which oxidative stress and microvascular damage play a role in pathogenesis, relative to the patients with non-DPN, diabetic patients without polyneuropathy, and healthy individuals. We aimed to evaluate the association between MHR and the decreased CMAP amplitude exhibited by patients with diabetic axonal polyneuropathy.

It is generally accepted that the pathogenesis of DPN is multifactorial: a combination of vascular and metabolic defects. The structural abnormalities of the endoneurial microvascular structure, defects in the vasoactive agents that regulate blood flow in the nerves, and changes to the autonomic innervation of the vascular structure of the nerves potentially contribute to the ischemia of the nerves. Deterioration of the polyol pathway, abnormalities in the lipid metabolism, increased end products of advanced glycation, and oxidative damage constitutes several of the metabolic factors associated with polyneuropathy.^[19]^

The change in the lipid metabolism is common in patients with diabetes and has been found to plays a key role in the pathogenesis of DPN.^[16]^ The hepatic lipase activity, increased by the insulin resistance, hydrolyzes the phospholipids in the LDL and HDL particles and therefore causes smaller and denser LDL particles and decreased HDL.^[20]^ Since the levels of LDL and triglyceride are usually high and the level of HDL is low in the patients with DPN, it was thought that there could be a relationship between these observations and the development of neuropathy. The altered plasma lipid profile induces neurovascular pathologies. The microangiopathic changes occurring in vasa nervorum associated with atherosclerosis can mediate the occurrence of polyneuropathy by affecting the feeding of peripheral nerves.^[21]^ Research has further demonstrated that the size of the LDL particle, which is a marker of atherogenic dyslipidemia, is also an independent risk factor for the occurrence of neuropathy.^[16,22]^ In addition, it was shown that increased levels of LDL and TG are associated with the quick progression of not only the final stage of renal failure and blindness but also peripheral neuropathy in patients with diabetes.^[23]^ Hypertriglyceridemia contributes to the occurrence and progression of DPN on account of its atherogenic potential^[24]^ and simultaneous changes caused by the Schwann cell myelin structure play a role in this.^[25]^ The anti-inflammatory, antioxidant, and anti-thrombotic effects of HDL was evinced by prior research;^[26,27]^ HDL behaves as an anti-atherogenic lipid by preventing the lipid transportation of macrophages with lipid loads to the arterial wall,^[28]^ and prevents the adhesion of monocytes to the arterial wall by inhibiting the endothelial expression of the adhesion molecules through its inhibition of CD11b activation.^[29]^

Changes to lipid metabolism are concurrent with low-grade inflammation in patients with diabetes. Past investigations have demonstrated that the levels of C-reactive protein, interleukin (IL)-1, IL-6, tumor necrosis factor-a, and inflammatory cytokines are high in patients with diabetes.^[30,31]^ The proinflammatory changes have a direct role in the pathogenesis of complications such as atherosclerosis, nephropathy, and neuropathy.^[32–34]^ The main type of cells that play a role in the occurrence of atherosclerosis, a lipid-guided inflammatory disease that induces proinflammatory cytokine secretion,^[35,36]^ is the monocyte. Hyperglycemia in diabetic patients activates them^[37]^: hyperglycemia causes oxidative stress, which further damages the nerve cells through lipid peroxidation, induction of the proinflammatory factors, waste of the cellular antioxidants, and pathologic activation of the repair mechanisms.^[38]^ It is therefore thought that MHR can indicate inflammation due to the proinflammatory effect of the monocytes, as well as the anti-inflammatory and antioxidant effects of the HDL cholesterol.

Previous studies have observed that the neutrophil/lymphocyte ratio (NLR), as an indicator of systemic inflammation, increases in patients with diabetes,^[39]^ diabetic retinopathy,^[40]^ nephropathy^[41]^— the latter 2 are complications of diabetes—and in diabetic patients with coronary artery disease.^[42]^ In a recent study conducted by Liu et al, the relationship between diabetic peripheral neuropathy and NLR was examined; it was observed that patients with high NLR exhibited a lower nerve conduction velocity and that such patients were more likely to develop polyneuropathy.^[43]^

As a new vascular inflammatory marker, MHR was investigated in a series of clinical studies: Kanbay et al demonstrated that an increase in MHR was associated with a decreased glomerular filtration rate in chronic kidney disease;^[10]^ Canpolat et al found that high MHR was associated with the slow coronary phenomenon.^[12]^; Kundi et al^[9]^ determined the relationship between MHR and the SYNTAX score in the context of coronary artery disease; Bolayir et al^[14]^ demonstrated that the high MHR was an independent risk factor for 30-day mortality in patients with ischemic stroke; and You et al^[7]^ showed that MHR was associated with the increased disability and mortality in patients with intracerebral hemorrhage.

The white blood cells and their subtypes, which are thought to be the potential predictors of clinical findings, morbidity, and mortality in a series of diseases, are commonly used markers of inflammation. IL-1, IL-6, IL-18, tumor necrosis factor-a1, interferon-1, transforming growth factor-1, and C-reactive protein are the inflammatory factors associated with the pathogenesis of diabetes.^[44–46]^ These circulating factors reflect low-grade chronic inflammation and are associated with the diabetic complications.^[47]^ However, their detection entails high costs, prohibiting their use in daily clinical practice. Laboratory indexes such as MHR, which features a lower cost and ease of measurement, have increasingly been adopted as indicators of systemic inflammation.

To the best of our knowledge, the present study is the first to assess MHR in diabetic patients with and without axonal polyneuropathy. We found that the MHR value was higher in patients with diabetic axonal polyneuropathy than in diabetic patients without polyneuropathy. The MHR values of both diabetic patients without polyneuropathy and patients with non-DPN were similar to those of healthy people. The group of patients with non-DPN consisted of individuals with chronic renal failure, those with malignancy who used chemotherapeutic agents, and patients with alcoholic neuropathy. In fact, the last group exhibited systemic as well. Despite this, the relatively higher MHR in patients DPN indicates that inflammation and oxidative stress were much more acute and suggests that the decreased HDL value may affect the pathogenesis of polyneuropathy more than we had expected.

Univariate and multivariate logistic regression analyses in the present study showed that age, disease duration, high levels of HbA1c, and elevated MHR values were independent predictors of DPN. Patients with DPN had a longer disease duration than did diabetic patients without polyneuropathy and their HbA1c levels were higher, that is, they exhibited worse glycemic control.

These findings are in line with the known pathogenesis of DPN. A longer duration of diabetes, poor glycaemic control, age-related neuronal attrition, and recognised risk factors have been implicated.^[4,15,48–50]^ Glycaemic control is the most important factor in the prevention and progression of DPN.^[51]^ High blood glucose level induces nerve damage due to vascular and metabolic deterioration.^[19]^ The study by Popescu et al^[52]^ showed that age is an independent predictor of DPN development in diabetic patients. A study has previously shown that a disease that leads to a subclinical impairment that lasts for decades results in affected neurons that are more prone to the consequences of age-related neuronal impairment.^[53]^ Therefore, age may be a factor that affects the development of polyneuropathy. As the person ages, the age-related neuronal deterioration will also increase.

Contrary to the study of Popescu et al,^[52]^ we found that the duration of the disease as a risk factor is independent of glycemic control. In their study, they claimed that in spite of good glycemic control, the risk of developing neuropathy is similar in patients with and those without diabetes. However, as mentioned above, the development of neuropathy in diabetic patients is not only due to hyperglycemia. Our results support that long-term disease duration is an independent predictor of polyneuropathy development in diabetic patients. Other than these factors, MHR as a marker of oxidative stress and inflammation is an independent predictor of DPN. This association remained significant even after adjusting for DPN-related factors and is important because it shows that inflammation and oxidative stress play an important role in the development of polyneuropathy in diabetic patients.

In this study, a significant positive correlation was found between HDL-C and posterior tibial nerve CMAP amplitudes; negative correlations between posterior tibial nerve CMAP amplitudes and MHR, and between the latter and both monocyte counts; and no correlation between CMAP amplitudes and HbA1c levels.

In brief, our results demonstrated that diabetic patients with higher MHR levels tend to have lower CMAP amplitudes and are more likely to develop polyneuropathy. MHR could, therefore, be used as a predictor of polyneuropathy in diabetic patients.

Our study was subject to several limitations. First, our sample was small. Second, other inflammation factors were not measured in this research. Third, this study was designed as a retrospective study. Future research should adopt a prospective design to establish the relationship between progression of polyneuropathy and MHR.

## 5 Conclusion

MHR, an indicator of systemic inflammation, is a valuable index of the modified lipid profile. It, therefore, has potential as a predictor of the pathogenesis of diabetic neuropathy.

## Acknowledgments

We would like to thank Editage (www.editage.com) for English language editing.

## Author contributions

**Conceptualization:** Gönül Vural.

**Data curation:** Gönül Vural, Şadiye Gümüşyayla.

**Investigation:** Gönül Vural, Şadiye Gümüşyayla.

**Methodology:** Gönül Vural.

**Project administration:** Gönül Vural.

**Writing – original draft:** Gönül Vural, Şadiye Gümüşyayla.

**Writing – review & editing:** Gönül Vural, Şadiye Gümüşyayla.

Gönül Vural orcid: 0000-0002-1245-7273.
